# Diagnostic Value of Ultrasonography and Magnetic Resonance Imaging in Ulnar Neuropathy at the Elbow

**DOI:** 10.5402/2012/491892

**Published:** 2012-07-24

**Authors:** Hormoz Ayromlou, Mohammad K. Tarzamni, Mohammad Hossein Daghighi, Mohammad Zakaria Pezeshki, Mohammad Yazdchi, Elyar Sadeghi-Hokmabadi, Ehsan Sharifipour, Kamyar Ghabili

**Affiliations:** ^1^Neuroscience Research Center, Tabriz University of Medical Sciences, Tabriz 51666-14756, Iran; ^2^Department of Radiology, Tabriz University of Medical Sciences, Tabriz 51656-65811, Iran; ^3^Department of Community Medicine, Tabriz University of Medical Sciences, Tabriz 51656-65811, Iran; ^4^Medical Philosophy and History Research Center, Tabriz University of Medical Sciences, Tabriz 51656-65811, Iran

## Abstract

*Aim*. To evaluate the diagnostic value of ultrasonography and magnetic resonance imaging (MRI) in patients with ulnar neuropathy at the elbow (UNE). *Methods*. We prospectively performed electrodiagnostic, ultrasonographic, and MRI studies in UNE patients and healthy controls. Three cross-sectional area (CSA) measurements of the ulnar nerve at multiple levels along the arm and maximum CSA(-max) were recorded. *Results*. The ulnar nerve CSA measurements were different between the UNE severity grades (*P* < 0.05). CSA-max had the greatest sensitivity (93%) and specificity (68%). Moreover, CSA-max ≥10 mm^2^ defined the severe UNE cases (sensitivity/specificity: 82%/72%). In MRI, ulnar nerve hyperintensity had the greatest sensitivity (90%) and specificity (80%). *Conclusion*. Ultrasonography using CSA-max is sensitive and specific in UNE diagnosis and discriminating the severe UNE cases. Furthermore, MRI particularly targeting at increased signal of the ulnar nerve can be a useful diagnostic test of UNE.

## 1. Introduction

 Ulnar neuropathy is the second most common cause of entrapment neuropathy after carpal tunnel syndrome [1]. The ulnar nerve could be trapped in every part of upper limb including wrist, elbow, or arm. Therefore, diagnosing the site of ulnar nerve injury is of great clinical and therapeutic importance. Ulnar neuropathy at the elbow (UNE) where the nerve passes through the cubital tunnel is the most common place of the ulnar nerve entrapment [2]. The diagnosis of UNE is based on obtaining medical history, clinical examination, and electrodiagnostic studies [1]. Due to false negative or nonlocalizing results of the electrodiagnostic studies, ultrasonography of the ulnar nerve has been recently recommended as an accurate noninvasive additional tool. However, ultrasonography still has no role in guidelines or practice parameters due to controversial results both in UNE patients and healthy individuals [3]. On the other hand, magnetic resonance imaging (MRI) is being increasingly used in the evaluation of ulnar neuropathy [4]. To date, few investigations have targeted at assessing the diagnostic value of MRI in UNE with debatable results [5–7]. To the best of our knowledge, the diagnostic value of both ultrasonography and MRI in UNE has not been hitherto investigated in a single study. Therefore, we aimed at evaluating the diagnostic value of ultrasonography and MRI in patients with UNE and also comparing the ultrasonographic and MRI alterations of the ulnar nerve in patients with different grades of UNE severity determined by electrodiagnostic studies.

## 2. Materials and Methods

### 2.1. Patients and Controls

 Between August 2010 and January 2012, twenty-nine elbows of 25 patients with a diagnosis of UNE were prospectively studied at Imam Reza Hospital, a university-affiliated teaching hospital. The study was approved by the local medical ethical committee of the Tabriz University of Medical Sciences. Informed consent was obtained from each subject in patient and control groups prior to the study. Inclusion criteria were the age of 15–65 years and presence of clinical findings and electrophysiological confirmation of UNE. The symptoms (numbness and paresthesia of the fourth and fifth digits of the hand, weakness or clumsiness of the hand muscles innervated by the ulnar nerve, and medial elbow pain) and signs (sensory loss in the area of the ulnar nerve and weakness of the ulnar innervated muscles) constituted the clinical diagnosis of UNE. Moreover, electrodiagnostic criteria for UNE were based on those proposed by the American Association of Neuromuscular & Electrodiagnostic Medicine (AANEM) (see below). Patients were excluded if any of they had history of polyneuropathy, acute trauma, previous trauma in the region of the elbow (including previous surgery), or symptoms of UNE more than one year.

 Thirty-five elbows of 23 healthy age-group-matched controls with no signs or symptoms of UNE or had systemic diseases associated with polyneuropathy were recruited for both ultrasonography and MRI studied.

### 2.2. Electrodiagnostic Studies

 Electrophysiological studies included needle electromyography (EMG), and nerve conduction studies of the median and ulnar nerves were performed in all patients. The studies were performed with Nicolet Viking IV electrodiagnostic system and TOENINNIES NeuroScreen Plus equipment. Ulnar sensory and motor nerve conduction studies were performed with the elbow flexed at 90°. To evaluate the motor conduction velocity (MCV) of the ulnar nerve, surface recording electrodes were located over the motor point of the abductor digiti minimi (ADM) and first dorsal interosseous (FDI) muscles. Surface stimulation was performed at the wrist, 4 cm distal to the medial epicondyle (below elbow) and 10 cm above this level (above elbow). The sensory conduction studies were done antidromically, stimulating at the wrist and recording from digit 4 or 5 for the ulnar nerve. The severity of UNE was defined as mild, moderate, and severe based on the following criteria [1].

- Mild involvement, presence of one of the following:
  - reduced motor conduction velocity (MCV) > 10 m/s across the elbow (segment below-above elbow), compared with the more distal segment (wrist-below elbow), from the muscle I dorsal interosseus (IDI) or Abductor Digiti Minimi (ADM), plus increased F-wave (compared with the unaffected side or normative value);
  - reduced amplitude of sensory nerve action potentials (SNAPs) at IV and/or V finger (compared with the unaffected side or normative value).
- Moderate involvement, presence of one of the following:
  - point 1 plus 2 of the previous grade;
  - motor conduction block from IDI or ADM at the elbow;
  - reduced amplitude of proximal compound muscle action potential (CMAP) across the elbow from IDI or ADM > 20 but <50% and/or abnormal EMG of ulnar hand muscles (acute and chronic denervation potentials) and/or SNAPs absence.
- Severe involvement, presence of one of the following:
  - complete motor conduction block alone across the elbow from IDI or ADM plus other abnormalities (point 3 of previous grade);
  - reduced amplitude of proximal CMAP across the elbow from IDI or ADM > 50%;
  - severe axonal involvement of ulnar nerve with SNAPs abnormalities and abnormal EMG of ulnar hand muscles (acute and chronic denervation potentials).

### 2.3. Ultrasonographic Evaluation

 In all the patients and controls, the ulnar nerve at the elbow was examined by the same radiologist blinded to the study using high-resolution ultrasonography (Medison multifrequency 7–14 MHz). The examinee sat and faced the operator with the examined upper limb and elbow flexed to 90°. Using automatic manual tracing method within the echogenic rim, four measurements including at the level of medial epicondyle cross-sectional area (CSA)-epi, 4 cm proximal to the medial epicondyle (CSA-prox), 4 cm distal to the epicondyle (CSA-dist), and the maximum cross-sectional area (CSA-max) of the ulnar nerve found between these points were performed in axial planes.

### 2.4. Magnetic Resonance Imaging Examination

 Axial, coronal, and sagittal T1-weighted and fat suppressed T2-weighted sequences in 3 mm slice thickness through the elbow joint were obtained from all patients and controls using a same 1.5 T magnetic resonance imager (Siemens, USA). The field of view was 10 cm centered at the medial epicondyle. A single observer who was blinded to the clinical, neurophysiologic, and ultrasonographic findings analyzed the MRI findings based on the signal intensity of the ulnar nerve, nerve compression, and nerve swelling. Increased signal intensity was qualitatively determined. The caliber of the ulnar nerve was pictured over its 10 cm field of view and any apparent (or qualitative) swelling or compression was quantitated using computerized measurements. The caliber of the ulnar nerve was deemed to be abnormal if there was greater than 20% increase (defined as nerve swelling) or decrease (defined as nerve compression) in cross-sectional diameter in relation to proximal and distal segments [7]. To reduce false positive rates, the ulnar nerve was assessed in 20 healthy controls who underwent a similar MRI study through the radiohumeral joint.

### 2.5. Statistical Analysis

 Data were presented as mean ± standard deviation or as median (interquartile range). All statistical analyses were performed with Statistical Package of Social Science (SPSS Inc., Chicago, IL) for Windows version 16. Chi-square or Fisher's exact tests were used to study the qualitative data, *t*-test for independent groups to compare quantitative variables, and Repeated Measures Analysis to evaluate the vital indices in both groups. The sensitivity and specificity of ultrasonography and MRI were studied by means of a receiver operating characteristic (ROC) curve. A *P* value less than 0.05 was considered statistically significant.

## 3. Results

 Twenty-nine elbows with UNE were studied.  Table 1 shows the baseline characteristics of the patients and controls included in this study. The median ulnar nerve CSA (square millimeters) at all the four levels (-prox, -epi, -dist, and -max) was significantly greater in UNE patients than in controls (*P* < 0.001, Table 1). The median ulnar nerve CSA at all the four studied levels (-prox, -epi, -dist, and -max) in the patient group was significantly different between the UNE severity grades (*P* < 0.05, Table 2).

**Figure 1:**
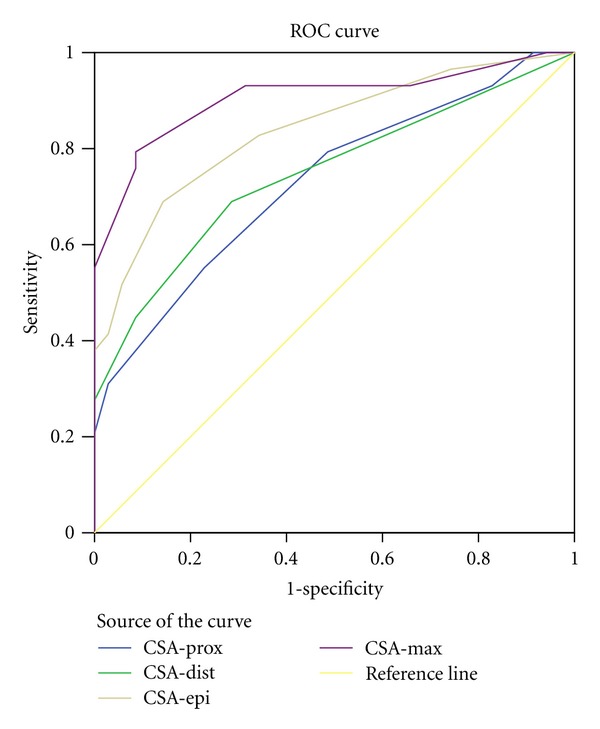
ROC curve for ultrasonographic measurements CSA: cross-sectional area.

 An ROC analysis provided the sensitivity and specificity of the ultrasonographic measurements. The CSA-max had the largest area under the curve and the greatest sensitivity and specificity (Table 3, Figure 1). Moreover, ROC analysis revealed the CSA-max cut-off point of 10 mm^2^ to define the severe UNE. This value yielded sensitivity and specificity of 82% and 72%, respectively. The same analysis did not result in significant cut-off values to define mild and moderate UNE (*P* > 0.05). 

 An MRI was performed in 21 UNE patients and 20 healthy individuals. Nineteen symptomatic patients (90.4%) and four (20%) normal volunteers had increased signal intensity of the ulnar nerve. In patients with UNE, ulnar nerve hyperintensity was followed by ulnar nerve swelling (9/21, 42.8%), combination of ulnar nerve hyperintensity and swelling (9/21, 42.8%), and ulnar nerve compression (7/21, 33.3%). In addition, ulnar nerve hyperintensity showed the greatest sensitivity (90%) and specificity (80%) than the other measured MRI variables (Table 4).

## 4. Discussion

 The present study revealed that the ulnar nerve CSA at all the four levels (-prox, -epi, -dist, and -max) was significantly greater in UNE patients than in the healthy individuals. These findings are similar to those of the previous studies [1, 8–11]. However, the median ulnar nerve CSA-max of both UNE patients and healthy individuals in the present study (9 and 5 mm^2^, resp.) is less than that of some similar studies [1, 9, 11–14]. The indicated values correspond to those of the similar study by Mondelli and colleagues [8]. In a recent review, Beekman et al. attributed this variation in normal CSA values of the ulnar nerve to different factors including selection of controls and use of the unaffected arm of UNE patients as control [3]. In the present study, we believe that low CSA-max values in the UNE patients might stem from higher number of the cases with mild UNE (45% of all patients) compared with moderate and severe cases of UNE. Nevertheless, we do not have an explanation for the low CSA-max values in the healthy individuals.

 In the present study, we found that the cut-off value of >5 mm^2^ for CSA-prox, -epi, and -dist had sensitivity of 72–82% and specificity of 51–71% in UNE diagnosis. The study by Bayrak and colleagues yielded relatively similar sensitivity and specificity of the CSA-prox and -dist; however, the cut-off values were higher (8 and 9 mm^2^) compared to those of our study [9]. In contrast to the present study, Bayrak et al. reported higher sensitivity and specificity (~82%) for CSA-epi cut-off value of >10 mm^2^ [9]. Nevertheless, lower sensitivity (46%) was indicated for CSA at the epicondyle (CSA-epi) cut-off value of >8.8 mm^2^ in the investigation by Mondelli and coworkers [8]. They attributed the low sensitivity of CSA-epi to recruitment of only electrophysiologically confirmed UNE patients, the presence of many cases of neurologically mild UNE, and the measurement of the CSA at a fixed point on an axial scan [8]. On the other hand, the diagnostic value of the CSA-max in UNE has been studied in several investigations. The present study showed that the cut-off value of >6 mm^2^ for CSA-max had sensitivity of 93% and specificity of 68% in UNE diagnosis. These findings are consistent with those of the study by Bayrak and colleagues (sensitivity/specificity: 95%/71%) for the cut-off value of >11 mm^2^ [9]. Other studies reported that the cut-off value of >8.3–10 mm^2^ for CSA-max had sensitivity of 88–100% and specificity of 88–98% in UNE diagnosis [1, 11, 15]. The diagnostic value of the CSA-max in UNE has been recently analyzed and readers are referred to the review by Beekman and colleagues [3]. As the aforementioned, lower cut-off values of the ulnar nerve CSA (-prox, -epi, -dist, and -max) in the present study might be attributed to the greater number of the cases with mild UNE compared with the moderate and severe cases of UNE as well as to the low CSA values in the healthy individuals.

 The present study also showed that the ulnar nerve enlargement, evaluated by CSA at all the four levels (-prox, -epi, -dist, and -max), was significantly linked to UNE severity. An association between the ulnar nerve and severity of nerve conduction abnormalities in UNE has been established in some previous studies [3, 16–18]. However only four investigations reported such an association between the CSA and UNE severity [1, 8, 9, 11]. To the best of our knowledge, the present study is the first investigation yielding the association between CSA at all the four levels (-prox, -epi, -dist, and -max) and UNE severity. Moreover, our study aimed at determining the CSA-max cut-off points discriminating between different grades of UNE severity. Accordingly, CSA-max cut-off point of 10 mm^2^ defined the severe UNE cases with sensitivity of 82% and specificity of 72%. Nevertheless, the present study failed to determine significant CSA-max cut-off values to define mild and moderate UNE cases. Among the previous similar trials, only one study was designed to find CSA cut-off points defining the severity of UNE [1]. Volpe and colleagues found two CSA-max cut-off values of >10 mm^2^ and >15 mm^2^ for the mild and moderate UNE diagnosis with a very good diagnostic performance [1]. Nonetheless, the cut-off point of >20 mm^2^ for the severe UNE showed sensitivity of 39% and specificity of 84% in their study [1]. Their findings are in contrast to ours in this regard.

 In our study, MRI analysis revealed that ulnar nerve hyperintensity had greatest sensitivity (90%) and specificity (80%). Similarly, high sensitivity of increased signal of the ulnar nerve (97%) in MRI was reported by Britz and coworkers [6]. In addition, isolated ulnar nerve swelling and combination of ulnar nerve hyperintensity plus swelling yielded a low sensitivity and high specificity in the present study. In contrast, considering the isolated swelling or combination of ulnar nerve hyperintensity plus swelling, MRI had excellent sensitivity and specificity in UNE diagnosis in the previous studies [5, 7, 19]. Altogether, it seems that sensitivity, specificity, and accuracy of the increased signal intensity of the ulnar nerve in this study are higher than those of the ulnar nerve size. This finding is consistent with that of the previous investigations by Britz et al. and Bäumer and colleagues [6, 19]. On the contrary, large number of healthy individuals (≥50%) with increased signal intensity of the ulnar nerve raised doubts about the diagnostic value of hyperintensity in MRI when compared with the ulnar nerve size or combination of increased size and signal intensity [5, 20]. As only one-fifth of the normal volunteers in our study had increased signal intensity of the ulnar nerve, we believe that increased signal of the ulnar nerve is more sensitive and specific than enlargement of the ulnar nerve in MRI.

 This study has certain limitations. A clear limitation is the small sample size for both patients and controls. Further similar studies with larger sample size would be valuable in definition of both ultrasonography and MRI cut-off points discriminating between different UNE severity grades. Furthermore, in some cases of the control group we used both arms as independent observations (artificial power increase) [3]. Moreover, we did not study underlying abnormalities and anatomical variations in patients with UNE. In addition, the diagnosis of UNE based on the electrodiagnostic studies is not an accurate “gold standard” method; current methods for diagnosing UNE are limited [5]. Also, our study did not focus on localizing or nonlocalizing abnormalities in the electrodiagnostic studies. On the other hand, the advantage of the current study is that this is the first investigation to assess the diagnostic values of both ultrasonography and MRI in UNE.

 In conclusion, as a useful complementary tool, ultrasonography of the ulnar nerve using maximum CSA (CSA-max) is both sensitive and specific in UNE diagnosis and discriminating the severe UNE cases from the mild and moderate grades. Furthermore, ulnar nerve MRI particularly targeting at the increased signal of the ulnar nerve can be a useful diagnostic test for evaluation of UNE, particularly in conjunction with clinical and electrophysiological data.

## Figures and Tables

**Table 1:** Baseline characteristics and CSA of the ulnar nerve at the elbow in the patients and controls.

Variable	Patients	Controls	*P* value
Age (years)	48.6 ± 13.5	42.1 ± 12.9	0.62
Gender (male : female)	16 : 9	15 : 8	0.85
Number of patients/examined elbows	25/29	23/35	—
Side affected: *n* (%)			NA
Right	10 (40%)	—	
Left	11 (44%)	—	
Bilateral	4 (16%)	—	
UNE severity: *n* (%)			NA
Mild	13 (44.8%)	—	
Moderate	8 (27.6%)	—	
Severe	8 (27.6%)	—	
CSA-prox (mm^2^)	6 (5–7)	4 (4-5)	<0.001
CSA-epi (mm^2^)	7 (5–12)	4 (3–5)	<0.001
CSA-dist (mm^2^)	5 (4–7)	4 (3–5)	<0.001
CSA-max (mm^2^)	9 (6.6–13.5)	5 (4–6)	<0.001

UNE: ulnar neuropathy at the elbow; CSA: cross-sectional area; prox: proximal; epi: epicondyle; dist: distal; max: maximum; NA: not available.

**Table 2:** CSA of the ulnar nerve at the elbow in different UNE severity groups.

Variable	Mild (*n* = 13)	Moderate (*n* = 8)	Severe (*n* = 8)	*P* value
CSA-prox (mm^2^)	5 (4–6)	6.5 (5.25–8.5)	6 (5.25–9.75)	0.03
CSA-epi (mm^2^)	6 (4.5–7.5)	6.5 (5.25–10)	13 (7.5–15.75)	0.04
CSA-dist (mm^2^)	4 (3–6)	5.5 (4.25–6.75)	9 (5–10.5)	0.01
CSA-max (mm^2^)	7 (6–9)	10 (7–14.37)	13.5 (10.5–16)	0.003

UNE: ulnar neuropathy at the elbow; CSA: cross-sectional area; prox: proximal; epi: epicondyle; dist: distal; max: maximum.

**Table 3:** ROC analysis of ultrasonographic measurements.

Variable	AUC	Cut-off value	Sensitivity	Specificity
CSA-prox (mm^2^)	0.73	5	79%	51%
CSA-epi (mm^2^)	0.83	5	82%	65%
CSA-dist (mm^2^)	0.74	5	72%	71%
CSA-max (mm^2^)	0.90	6	93%	68%

ROC: receiver operating characteristic; CSA: cross-sectional area; prox: proximal; epi: epicondyle; dist: distal; max: maximum; AUC: area under the curve.

**Table 4:** Sensitivity and specificity of MRI measurements.

Variable	Sensitivity	Specificity	*P* value
Ulnar nerve hyperintensity	90%	80%	<0.001
Ulnar nerve swelling	42%	100%	0.01
Ulnar nerve hyperintensity and swelling	42%	100%	0.01
Ulnar nerve compression	33%	100%	0.06

MRI: magnetic resonance imaging.
